# Cortical Cartography: Mapping Arealization Using Single-Cell Omics Technology

**DOI:** 10.3389/fncir.2021.788560

**Published:** 2021-12-10

**Authors:** Patricia R. Nano, Claudia V. Nguyen, Jessenya Mil, Aparna Bhaduri

**Affiliations:** Department of Biological Chemistry, University of California, Los Angeles, Los Angeles, CA, United States

**Keywords:** cortex, development, single-cell, multi-omic, regions

## Abstract

The cerebral cortex derives its cognitive power from a modular network of specialized areas processing a multitude of information. The assembly and organization of these regions is vital for human behavior and perception, as evidenced by the prevalence of area-specific phenotypes that manifest in neurodevelopmental and psychiatric disorders. Generations of scientists have examined the architecture of the human cortex, but efforts to capture the gene networks which drive arealization have been hampered by the lack of tractable models of human neurodevelopment. Advancements in “omics” technologies, imaging, and computational power have enabled exciting breakthroughs into the molecular and structural characteristics of cortical areas, including transcriptomic, epigenomic, metabolomic, and proteomic profiles of mammalian models. Here we review the single-omics atlases that have shaped our current understanding of cortical areas, and their potential to fuel a new era of multi-omic single-cell endeavors to interrogate both the developing and adult human cortex.

## Introduction

Fundamental aspects of cognition rely on the precise organization of the cerebral cortex into distinct areas, each with unique cytoarchitectural structures and physiological roles that have been examined for over a century (Brodmann, 1909; von Economo and Koskinas, 1925; Bayer and Altman, 1991; Hof and Nimchinsky, 1992; DeFelipe et al., 1999; Elston, 2003). These areas process complex functions through a hierarchical network. For example, optogenetic studies have shown the roles of specific cortical areas at various steps of decision-making processes, such as the requirement of the barrel cortex as mice first sense object presence *via* their whiskers. The subsequent processing between sensation and action is then driven by the anterolateral motor cortex (ALM) (Guo et al., 2014). Coupling the optogenetic control of neuronal activity with live calcium imaging has enabled the tracking of information as it flows through these areas. For example, as mice move from preparing for a motor task to actual movement, neuronal activity travels from confinement within ALM to superficial ALM layers and finally to other motor cortical regions (Chen et al., 2017). Similar work tracking areal relationships within the mouse visual cortex has shown the presence of highly connected areas such as the primary visual cortex (V1) and the posteromedial (PM) and lateromedial (LM) areas. Each of these areas is specialized toward processing specific visual cues (D’Souza et al., 2016). Inter-areal communication is hierarchical, with areas located deeper in the brain harboring the greatest number of connections. Many of these connections are comprised exclusively of excitatory inputs that activate both excitatory and inhibitory neurons to temper the information flow from multiple stimuli (D’Souza et al., 2016). In this way, cortical areas provide a crucial modular network wherein various forms of information are processed by specialized areas and collected into higher-order regions to make complex decisions.

Accordingly, a variety of neurological disorders impact sections of this modular network, resulting in area-specific phenotypes. Autism spectrum disorder is characterized by cellular changes in frontal areas (Carper and Courchesne, 2005), such as increased neuronal density in the anterior cingulate cortex (Simms et al., 2009), disrupted morphology of neuronal minicolumns in the frontal and anterior cingulate cortices (Buxhoeveden et al., 2006; Casanova et al., 2006), and abnormal microglial activity in the prefrontal cortex (PFC) (Morgan et al., 2010). Aberrant prefrontal areas are also a characteristic of schizophrenia, including decreases in the gray matter of prefrontal, cingulate, and orbital frontal cortices (Ellison-Wright et al., 2008; Nestor et al., 2010). This is also accompanied by white matter dysregulation in the anterior cingulate cortex (Manoach et al., 2007), and disrupted connections between the PFC and the temporal and parietal lobes (Zhou et al., 2007; Konrad and Winterer, 2008; Yoon et al., 2013). These phenotypes correspond to a decrease in behavioral performance and clinical status (Lawrie et al., 2002; MacDonald et al., 2005; Meyer-Lindenberg et al., 2005; Eack et al., 2013; Yoon et al., 2013). In contrast, symptoms of Huntington’s disease are correlated with the selective loss of pyramidal neurons in the primary motor and premotor cortices and, to a lesser extent, superior frontal and parietal areas (Macdonald and Halliday, 2002; Thu et al., 2010). Understanding cognition in both normal and disease states therefore relies on a comprehensive understanding of how highly specialized areas are patterned in the cerebral cortex.

While signaling mechanisms and pathways that push neural stem cells to a specified areal fate have not been fully evaluated in the human cortex, a hypothesis for the emergence of neuronal diversity in the mature nervous system has been delineated in *Drosophila* (Sen et al., 2019). In this model, spatial transcription factors (STFs) and temporal transcription factors (TTFs) create the diversity of neuroblasts seen in *Drosophila*. Spatial patterning generates initial neuroblast identities, while temporal patterning generates different progeny within each neuroblast lineage. These observations serve as a model and an aspiration for the field of mammalian cortical development, for which known transcriptional programs are currently insufficient to similarly describe the full swath of cell type heterogeneity.

Recent efforts to probe mechanisms of human cortical arealization have taken advantage of single-cell omics technologies to map the cortex. These atlas-scale characterizations of the cortex have enabled its parcellation at the molecular level, characterizing gene regulatory mechanisms and their integration with chromatin accessibility, electrophysiology and other functional modalities at high resolution. In the following sections, we review how these advancements have refined our understanding of the boundaries between cortical areas, the mechanisms of cortical patterning, and the need for increasingly multimodal “omic” analyses to deepen our molecular understanding of the cortical hierarchical network.

## Molecularly Defining Cortical Areas *via* Transcriptomic Profiles

For the past decade, transcriptomic studies have sought to resolve the molecular networks that generate area-specific functions. Early efforts to profile the cortex include microarray analyses of bulk human neocortical tissue throughout life (Kang et al., 2011; Pletikos et al., 2014), which quantitatively compared gene networks in 11 neocortical areas across 15 ages from embryonic stages to late adulthood. Hierarchical clustering of these areal transcriptomes at multiple timepoints revealed that distinct gene expression patterns were present in a variety of areas in the developing cortex. However, later ages saw an increasing similarity across all the cortical areas, with the exception of the medial PFC and V1 at opposing ends of the cortex (Kang et al., 2011; Pletikos et al., 2014). These microarray analyses support a model in which the transcriptional divergences between cortical areas during development are dampened into more subtle variations in the adult.

The emergence of bulk RNA-sequencing (bulk RNA-seq) enabled a reevaluation of this relatively homogenous transcriptional identity in the adult human cortex with an unbiased approach of greater specificity, sensitivity, and dynamic range. Interestingly, the majority of such adult bulk RNA-seq profiles did not find striking differences between the cell types and transcriptomes of cortical areas (Khaitovich et al., 2004; Roth et al., 2006; Hawrylycz et al., 2012, 2015; Kelley and Oldham, 2015; Xu et al., 2018). Conclusions from these assays therefore supported the theory that developmental differences in gene expression become increasingly ordered into transcriptionally similar, but functionally distinct, cortical areas. However, bulk RNA-seq averages gene expression across entire cell populations and is therefore unable to resolve the variability in transcript distribution among individual cells. In particular, gene expression may appear more uniform when averaged due to the fact that major cell type populations, such as glutamatergic and GABAergic neurons, are present across the majority of areas (Yamawaki et al., 2014). Resolving the true transcriptomic boundaries of cortical areas therefore relies on an RNA-sequencing method that probes identity at the cellular level.

Single-cell RNA-sequencing (scRNA-seq) has provided the novel ability to generate cell types and clusters based on the transcriptomic profiles of individual cells. The increased resolution of single-cell analyses has identified subtle transcriptomic differences between cell clusters, even those within the same broader cell class (i.e., excitatory neuron, inhibitory neuron, astrocyte, etc.). Consequently, these atlases have identified unique profiles within and between adult cortical areas that were underappreciated by microarrays or bulk sequencing (Li et al., 2018; Zhu et al., 2018; Yao et al., 2021). For example, recent single-cell transcriptomic analyses in the adult mouse neocortex have disentangled nuanced differences between excitatory, glutamatergic neurons and GABAergic interneurons. These atlases have revealed that interneuron transcriptomes are largely homogenous across the cortex (Tasic et al., 2018; Yao et al., 2021). Similarly, broadly defined subclasses of excitatory neurons were shared between most cortical areas (Yao et al., 2021). However, within these classes of excitatory neurons exist clusters of cells with slightly distinct transcriptional profiles that are unique to a subset of neighboring cortical areas (Yao et al., 2021). These subtle differences give rise to gradual transcriptomic shifts across both the anterior–posterior and the mediolateral axes, rather than discretely defined transcriptional identities (Yao et al., 2021). This nuanced transcriptomic variation also corresponds to differences in the expression of areal marker genes. For example, the expression of the putative transcriptional repressor, *Tshz2*, is enriched in the medial poles of the adult murine cortex (Yao et al., 2021). However, in most glutamatergic subtypes, *Tshz2* expression is still detectable across multiple cortical areas (Yao et al., 2021). In addition, these scRNA-seq profiles have identified gradual transcriptomic differences between the layers of the adult mouse cortex and revealed the expression of laminar marker genes such as *Rorb* (layer 4) in multiple layers (Yao et al., 2021).

Initial single-cell analyses of the PFC and V1 in the developing human cortex indicated that stark transcriptomic differences between the excitatory neurons of these two areas exist at early developmental timepoints (Nowakowski et al., 2017). Individual genes also displayed discrete area-specific patterns. For example, while PFC neurons displayed a co-expression of the subcortical projection marker *BCL11B* and the intracortical projection marker *SATB2*, expression of these genes did not colocalize in V1 neurons (Nowakowski et al., 2017). More recent developmental and adult scRNA-seq studies have refined this theory, as the comparison of more cortical areas and the integration of molecular and connectivity profiles point to a continuum of cell types both within and across adult cortical areas. These studies have revealed that in both the developing and adult cortex, neighboring cortical areas have overlapping transcriptomic features that become increasingly distinct with greater spatial distance (Bhaduri et al., 2021; Yao et al., 2021). For example, the posterior-high to anterior-low gradient marker gene *NR2F1* is expressed at higher levels and in more cells in the posteriorly located V1 compared to the PFC (Bhaduri et al., 2021). However, *NR2F1* expression is also enriched in the temporal and parietal lobes proximal to V1 and gradually decreases along the posterior to the anterior axis (Bhaduri et al., 2021). Therefore, functionally discrete areas may arise from subtle transcriptional differences that lay the foundation for phenotypic differences found among the cortex.

Recently, spatial transcriptomics have expanded upon traditional RNA-seq profiles by visualizing how the localization of transcriptomically diverse cell types could delineate functionally distinct cortical areas. These techniques apply imaged-based sequencing of fluorescently tagged probes to determine the subcellular localization of individual mRNA transcripts. Spatial transcriptomics thus generates an inferred single-cell transcriptome based on the expression profile of genes confined within cellular boundaries (Ke et al., 2013; Lee et al., 2014; Chen et al., 2015). While spatial transcriptomics has yet to be widely applied toward human cortical tissues, protocols that maximize the number of genes probed per cell (Eng et al., 2019; Zhang M. et al., 2021) or integrate high-throughput sequencing strategies (Ståhl et al., 2016; Rodriques et al., 2019; Srivatsan et al., 2021) show promise in capturing the increased complexity of human cortical gene expression. Advancements in methods to efficiently process such imaging and transcriptomic profiles (Zhuang, 2021) may also bring spatial transcriptomics closer to the increased computational demands of profiling larger, more complex human cortical tissue.

Several spatial transcriptomic profiles in the adult mouse neocortex have begun to clarify how area-specific transcriptional identities generate cytoarchitectural variation. For example, an adult mouse study comparing the expression of 160 genes in the V1 versus medial PFC (Wang et al., 2018) showed that both areas share a general pattern in which inhibitory neurons are sparsely distributed and excitatory neurons are loosely segregated into layers. However, subtypes of excitatory neurons can also show area-specific distributions: a few cell types are lacking or reduced in the medial PFC relative to V1, but the medial PFC contains new cell types in layers 5 and 6. Spatial transcriptomic datasets can also reveal how cell populations display differences across both local neighborhoods and across wider regions (Codeluppi et al., 2018; Eng et al., 2019). Such profiles of the adult mouse neocortex (Zhang et al., 2020) showed the presence of individual neighborhoods within cortical areas that are composed of their own complex mix of cell types, with these communities increasing in complexity in the deeper cortical layers. Future applications in human cortical areas may therefore reveal how molecularly defined cell types interact with each other to build areas with unique cellular neighborhoods and, ultimately, specialized functions.

Other versions of the spatial transcriptomic approach have conducted unsupervised or hierarchical clustering analyses to define how more refined cell types are organized throughout the adult mouse cortex (Codeluppi et al., 2018; Eng et al., 2019; Zhang et al., 2020). Importantly, these spatially defined clusters displayed high correlation with those determined in traditional single-cell transcriptomic datasets in the adult mouse cortex (Yao et al., 2021). For example, in spatial transcriptomic profiles of the motor and primary somatosensory areas in the adult mouse, the majority of neuronal cell types were evenly distributed along the anterior–posterior axis while a small group of glutamatergic subtypes were enriched at either anterior or posterior pole (Zhang et al., 2020).

**FIGURE 1:**
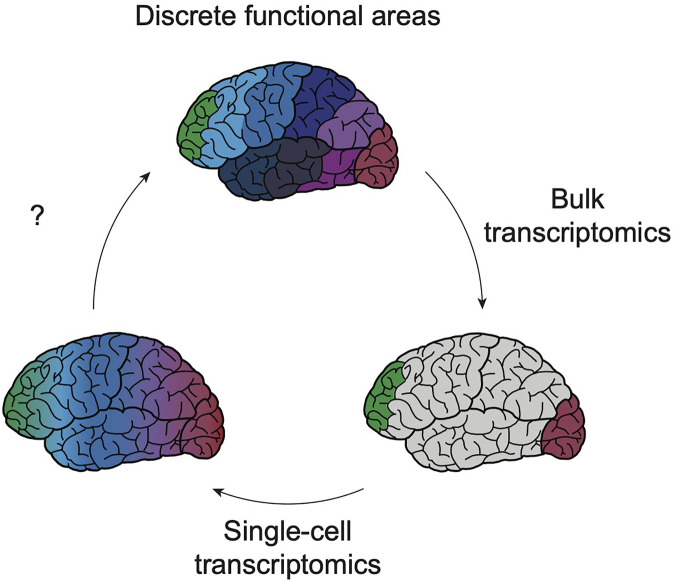
Exponential resolution of transcriptomic profiling shaped understanding of cortical boundaries. Early profiling efforts of bulk tissue (bottom right schematic) from various cortical areas have suggested that, with the exception of the poles of the cortex (green, red) the majority of cortical areas share a largely consistent transcriptomic profile (gray). Single-cell transcriptomics has sought to increase the resolution of these profiling techniques, enabling the detection of transcriptomic profiles of cell type-specific areal signatures and of rare cell types (bottom left). However, these next-generation datasets continue to show the subtlety of transcriptomic heterogeneity between cortical areas, suggesting that gene expression differences between these regions exist across a continuum. Future work using human model systems will continue to refine our understanding of how these blurry boundaries ultimately yield discrete functional properties (top).

**FIGURE 2:**
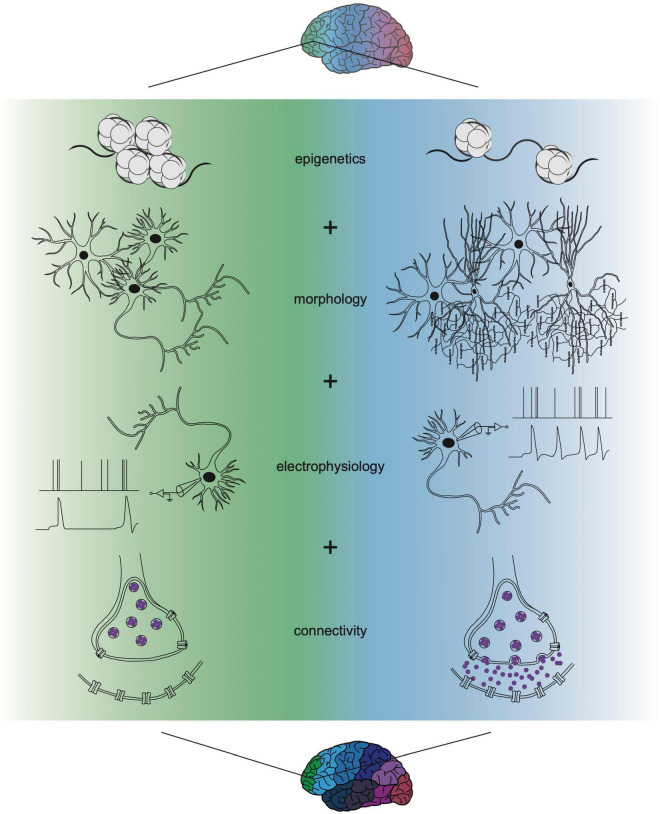
Multi-omic single-cell approaches refine understanding of cortical cell function. Novel strategies applying the integration of transcriptomics with profiles of epigenetics, morphology, electrophysiology, and connectivity are refining areal differences in cell type above what can be elucidated from transcriptional identity alone. Transcriptional differences may initiate and propagate the onset of these other modalities, together resulting in discrete cell type and unique areal function. Recent multi-omic technological advances have already improved characterization of cell types and the field will continue to integrate more properties as we fully understand cortical cell biology. The identification of multi-omic states that integrate each of these functions will be needed to determine how such phenotypic distinctions generate stark functional specializations for cortical areas.

Though the past decade has seen advancing levels of depth and resolution in transcriptomic profiling, a consistent conclusion can be drawn from most of these datasets: the subtle differences in transcriptional identity and cell type “neighborhood” composition between cortical areas fails to mirror the discrete functional differences between cortical areas found in the adult cortex (Figure 1). How such graded molecular differences give rise to stark phenotypic diversity throughout the cortex remains unclear. One hypothesis could be that before the onset of maturation and functional properties (i.e., morphology, electrophysiological activity, and connectivity), gene expression is a primary modality of indicating cell identity. Over time, the emergence of these other properties allows for dynamic gene expression programs to iteratively specify these functions and then fade away. This model could explain how distinct area-specific functions arise despite subtle transcriptional differences (Figure 2). It also underscores that, while there is room for improved sampling density of both developing and adult cortical areas, transcriptomics is insufficient for understanding cortical arealization. Transcriptomic analyses alone indicate that current definitions of cortical areas may be too rigid. However, to determine whether cortical areas truly lie on a continuum or exist as discrete zones, molecular profiles require integration with other functional characteristics.

## The Mechanisms of Cortical Arealization Stem From Both Intrinsic and Extrinsic Signaling

Animal model studies have implicated roles for both intrinsic cortical area identities and extrinsic signals from neighboring brain regions in developmental cortical patterning and areal refinement (O’Leary et al., 2007; Cadwell et al., 2019). These experiments have revealed the significance of morphogenetic gradients such as bone morphogenetic proteins (BMPs), Wnts, Sonic hedgehog (Shh), and fibroblast growth factor (FGF) in early patterning of the developing cortex (O’Leary et al., 2007; Cadwell et al., 2019). Many of these seminal studies provided detailed transcription factor (TF) maps but utilized rodent or chick embryos and occurred before the emergence of next-generation sequencing. Due to these limitations, it remains unclear how molecular cues can guide morphogenetic gradients to eventually give rise to the distinct cortical areas in the adult human brain. Single-cell transcriptomic studies have not solved this issue, but they have established profiles of the developing and adult human cortex. Thus, while scRNA-seq has enriched our understanding, perturbations of human models will be necessary to clarify our conception of human brain-specific developmental mechanisms.

Mechanistic studies are particularly important in determining the relationship between cortical morphogenic gradients and extrinsic developmental cues. While it is known that extrinsic cues work in concert with intrinsic signaling to refine cortical regionalization during development, very little is known about the transcriptional impact of extrinsic signals on the cortex (Nakagawa, 2019). It has been established that the thalamus plays a particularly vital extrinsic role, as it forms reciprocal projections with the cortex during early brain development. For example, rodent studies have demonstrated that thalamocortical afferents may control cortical area size by promoting division of cortical neural progenitor cell populations (Dehay et al., 1991, 2001; Reillo et al., 2011). Beginning at embryonic timepoints, thalamocortical inputs also instruct the maturation and migration of cortical interneurons that originate in the medial ganglionic eminence and populate the cortex (Sugiyama et al., 2008; De Marco García et al., 2015; Marques-Smith et al., 2016; Tuncdemir et al., 2016; Zechel et al., 2016; Larsen et al., 2019). Additionally, glutamate release from thalamocortical afferents promotes morphological development of layer neurons in the postnatal primary somatosensory cortex (Narboux-Nême et al., 2012; Li et al., 2013). These cell identity oriented molecular cues culminate in laminar and areal fates, as thalamocortical afferents (Dehay et al., 1991, 2001; Reillo et al., 2011) can also instruct specification toward sensory areal identity in neocortical postmitotic neurons (Chou et al., 2013; Vue et al., 2013; Pouchelon et al., 2014) and regulate neurogenesis and laminar fates in sensory cortex (Monko et al., 2021). Rodent studies have therefore improved our understanding of thalamocortical circuit assembly and refinement across brain regions. This work has focused particularly on sensory systems such as the dorsal lateral geniculate nucleus (dLGN) that processes and relays input from the retina to the primary visual cortex (Kalish et al., 2018; Bakken et al., 2021). However, the role of thalamocortical afferents in neocortical development is not limited to sensory areas. For example, defects in interneuron migration were also found in medial PFC (Larsen et al., 2019), heavily suggesting that thalamocortical regulation of cell identity and arealization is universal in the neocortex. It is apparent that transcriptomic profiling of human thalamic tissue and perturbation in culture systems are necessary to distinguish how intrinsic and extrinsic cues generate diverse cell types, areas, and hierarchical networks with discrete functional differences.

Molecular signals within the cortex and those derived from extrinsic subcortical sources likely collaborate or compete with one another to generate molecular distinctions between cell types through developmental time. This diverse tapestry of transcriptional profiles may then underlie the presence of both discrete differences and continuous gradients of variation found between human cortical areas (Nowakowski et al., 2017; Li et al., 2018; Zhu et al., 2018; Bhaduri et al., 2021; Yao et al., 2021). This mixture of discrete and graded transcriptomic boundaries may be dynamic throughout development: inter-areal transcriptional states exhibit robust divergence prenatally, become more similar in infancy and childhood, and exhibit robust differences again in adolescence and onward (Li et al., 2018; Zhu et al., 2018). More refined single-cell transcriptomic datasets encompassing a greater number of cells, areas, and individuals showed that areal identities are also highly dynamic throughout neuronal differentiation, even though they remain most distinct at the PFC and V1, located at opposite poles of the cortex (Bhaduri et al., 2021). Taken together, these datasets reinforce the hypothesis that subtle but dynamic gene expression gradients in prenatal cortical progenitors become increasingly organized during cortical differentiation to give rise to areas with discrete neuronal subtypes and functionality.

These comparisons across developmental time highlight challenges for linking distinct gene regulatory networks to the discrete functions of the adult cortex. Understanding the emergence of areal network modules will ultimately require integration of other omic modalities with gene expression measurements. Integration of these modalities, such as morphology, connectivity, and epigenetics, will clarify the mechanistic origins of cortical areas and may revise what criteria defines an area (Figure 2). For example, exploring the intersection of transcriptomics and electrophysiology can test the hypothesis that dynamic gene expression over development may predict mature electrophysiological signatures. Additionally, studying relationships between transcriptomics and corticothalamic connectivity can elucidate whether transcriptional state or circuit connectivity primarily drives functional differences. Several cross-institutional research initiatives, such as the Human Brain Atlas and the NIH BRAIN Initiative, are currently seeking to broaden investigation of cortical and subcortical brain regions, including the thalamus. These enterprises that aim to elucidate the profiles and functions of understudied structures will not only increase area-specific knowledge but will also deepen our understanding of how relationships between them may facilitate discrete areal functions across development.

## Bridging Molecular and Functional Characterization of Areas With Multi-Omic Analyses

Mechanistic studies in animal models provide a wealth of hypotheses regarding how parameters beyond transcriptional differences influence cortical arealization in humans. In a recent study of *Drosophila* (Sen et al., 2019), the authors explored two hypotheses on the integration of spatial transcription factors (STFs) and temporal transcription factors (TTFs) during development in neuroblasts: (1) independent specification, where STFs and TTFs bind to genomic regions independently from one another to generate diverse neuronal lineages or (2) sequential specification, where STFs influence the chromatin landscape in each neuroblast resulting in neuroblast-specific TTF DNA-binding. The results supported a sequential specification hypothesis in which STFs influence the chromatin landscape, opening specific chromatin domains for TTFs to bind, thereby generating diverse neuronal populations in each neuroblast lineage. Future work may apply this sequential specification hypothesis as a framework to study the roles of modular gene regulatory networks in generating neuronal diversity observed across human cortical areas.

These *Drosophila* studies also exemplify the reliance of well-characterized TF-mediated arealization events on epigenetic regulation. In particular, chromatin landscape modifications serve to open domains and enable the binding of TFs including *Emx2*, *Pax6*, *COUP-TFI*, and *Sp8* (O’Leary et al., 2007). A variety of mechanisms and molecular factors may influence how these TFs can act differently across the cortex. For example, mouse *Pax6* has been found to interact with chromatin modifiers to maintain the neural progenitor pool and allow for upper layer neuron specification (Ypsilanti and Rubenstein, 2016). Recent work in mice has shown that terminal differential factors such as *Cux1, Cux2*, and *Satb2* can also specify ultimate upper layer fate, and that upper layer cells inherit the transcriptional landscape of their mothers (Heavner et al., 2020). These findings strongly suggest that epigenetic state, often influenced by TF expression, may bolster our understanding of cell type and how their developmental trajectories give rise to cortical arealization. Thus, the parallel or simultaneous sampling of these modalities is essential.

While epigenetics may be influenced by TF expression, epigenetic profiles also influence gene expression profiles. Previous studies have elaborated on the role of enhancers in tissue-specific gene expression and examined the genome-wide activation of enhancers in mouse tissues. These studies tied the utilization of these enhancers to designated functions and regulatory pathways in individual tissues across developmental stages (Nord et al., 2013). Enhancer activity is regulated in tight temporal and spatial windows, further supporting their role as a marker of cell type and location. In addition, enhancers have been shown to add greater specificity in activating certain cellular populations than their associated genes and contributing to the complexity arising in the developing forebrain (Visel et al., 2013). Screening of enhancer activity across multiple mouse cortical subregions identified several region-specific enhancers, supporting a specified, localized activity role in these regions (Blankvoort et al., 2018). It is important to note that not every enhancer will correlate to a specific cell type, as several enhancers may be active across cell types. However, combinations of different enhancers can generate a unique epigenetic profile, resulting in a unique cell identity. These data further support the imperative role of enhancers in cortical development. While enhancers may appear to play a more direct role in cell fate than transcriptomics, enhancer activity remains subject to regulation by TFs, as in pallial protodomains forming distinct cortical regions (Pattabiraman et al., 2014). Thus, we see an interplaying loop between the epigenome and transcriptome during development that drives cellular diversity in cortical arealization.

Single-cell-based epigenetic characterization of cell types has recently enabled the same single-cell advantages of transcriptomics, but with access to TF-relevant regulatory regions. Single-cell transposome hypersensitive site sequencing can generate maps of TFs, regulatory elements, and DNA accessibility to characterize extensive cellular diversity in the human cortex and cerebellum (Lake et al., 2018). Single-cell DNA methylation analysis has been shown to identify excitatory and inhibitory neuron subtypes (Luo et al., 2017), and single-nucleus methylation assays have led to the construction of taxonomies defined by signature genes, regulatory elements and TFs in 45 regions of the mouse cortex, hippocampus, striatum, palladium, and olfactory areas (Liu et al., 2021). This comprehensive assessment of the mouse brain cell epigenome elucidates the regulatory landscape that supports cell fate specification, while also revealing repetitive use of these regulators found in excitatory versus inhibitory neurons to further distinguish subtypes. Furthermore, by considering the predicted transcriptional profile specialized to each cell type, linking *cis*-regulatory elements to putative genes expressed in these cell types allow for high specificity of spatial distribution of such cell types (Li et al., 2021). Single-cell-based epigenetics such as single-nucleus DNA methylation sequencing can also be combined with lineage tracing tools to provide unique epigenetic signatures of neuron projections relative to their laminar and regional location (Mich et al., 2021; Zhang Z. et al., 2021). Additionally, the epigenome can often predict future transcriptional identities. Recent work exploring mouse cortical development has highlighted early specification events based upon the epigenome (Preissl et al., 2018). In the human, integration of both the single-cell epigenome and transcriptome showed that epigenetic distinctions between PFC versus V1 were apparent in intermediate progenitor cells, although transcriptional definitions of these areas emerged only after these cells differentiated into newborn neurons (Ziffra et al., 2021). Advancing technology to not only sample the epigenome at single-cell levels, but also to integrate it with single-cell gene expression assays, poses a unique opportunity to understand the regulation that precedes the emergence of gradient gene expression patterns, and may add an additional layer to characterizing how discrete functions across cortical regions emerge.

Several studies have leveraged epigenomic profiles in populations of interest to identify and characterize cell type specific *cis*-regulatory regions. One study in mice explored enhancers to identify key regulators of tissue-specific developmental gene expression trajectories (Nord et al., 2013). Another leveraged adeno-associated viruses (AAVs) and synthetic promoters to generate tools for cell type specific interrogation of glial populations across mice, non-human primates, and human cells (Juttner et al., 2019). Recent work has also used these open chromatin regions to distinguish between the developmentally elusive parvalbumin (PV) and vasoactive intestinal peptide-expressing (VIP) interneurons (Vormstein-Schneider et al., 2020). Single-nuclei ATAC-sequencing has expanded these technologies to more cell types and across species, enabling high-throughput discovery of cell type-specific expression of marker genes and corresponding AAV tools (Mich et al., 2021). Epigenomic profiling therefore not only provides crucial insights into other modalities for cortical arealization, but can advance the development of tools that can dissect the mechanisms of areal patterning.

Beyond epigenetics, the advent of multi-omic approaches that enable simultaneous sampling of multiple cellular properties are elucidating the correlations between gene expression, morphology, physiology, and connectivity (Figure 2). Patch-seq is an example of a multi-modal technique that combines patch-clamp recording, biocytin staining, and scRNA-seq (Cadwell et al., 2016, 2017). A recent patch-seq study in the adult mouse motor cortex supports a lack of discrete boundaries in the adult mouse primary motor cortex, providing evidence of a continuum of correlated transcriptomic and morpho-electrical clusters present within families of cells (Scala et al., 2020). Consistent with these observations, recent comprehensive work has shown that while gene expression can be correlated to a cell’s electrophysiology and morphology, each additional parameter adds slightly more nuanced variation (Nandi et al., 2020; Haghighi et al., 2021). As tools emerge to interrogate individual cell connectivity and merge them with neuronal subtypes defined in other contexts, heterogeneity has been identified within “subclasses” (Campagnola et al., 2021). These findings highlight gaps in our understanding of the complete set of properties that define how a cell functions. Future advancements in patch-seq applications have the potential to ascertain relationship dynamics between transcriptomics and electrical activity. Perhaps electrophysiological trajectory is inversely associated with gene expression, in that minute electrophysiological differences during infancy may become increasingly pronounced as humans develop toward adulthood.

These multi-omic approaches are slowly adding color to our understanding of which properties define a cell type and confer unique functions across cortical areas. However, some combinations remain technically difficult and are only beginning to be established. One such example is the intersection of transcriptomics and metabolomics, where burgeoning work already highlights the potential of this approach. Altered metabolic profiles may characterize distinct cell types with different functional energy requirements or that are in different cell states. For example, when neurons are excited, they change their metabolic state and rely on astrocyte lactate production to fulfill their energy needs (Maffezzini et al., 2020). Other subtler examples of similar shifts associated with cell type or state may improve our understanding of the functional role distinct cell types play across cortical areas. As reviewed in Lopes et al. (2020), there are also other combinations of multi-omics approaches that could further distinguish cell types’ lineage trajectory analyses, including the sampling of chromatin conformation.

Another single-cell omic technology that has remained elusive is single-cell proteomics. The proteome is particularly exciting because it has been well documented to not perfectly correlate to the transcriptome (Wang et al., 2019). However, various technological and sample preparation challenges have kept this technology at bay, or at least have limited the throughput of this method. These challenges and potential ways around them are well outlined in Buxhoeveden et al. (2006) and Labib and Kelley (2020), and recently published methods suggest that single-cell proteomics may be on the brink of resolving some of these challenges (Schoof et al., 2021). Recent multi-modal single-cell approaches, such as trimodal transcriptomic, epitope, and (chromatin) accessibility sequencing (TEA-seq), enable sampling of proteomic epitopes as well as the transcriptome and epigenome (Swanson et al., 2021). Such tools promise to extend bulk correlations to cell type-specific relationships. These insights could then tease apart if all cells have the same relationships between transcription and translation, as well as how the epigenome may have regulatory impacts upon this process. It is likely that every modality of the cell contributes to or is impacted by these specification events, and metabolomic and proteomic characterization of cortical areas during development may illuminate their specific role.

Each multi-omic analysis has shown that there are subtle temporal or identity differences between gene expression and epigenetics, electrophysiology, morphology, or connectivity. Although each of these parameters appears to emerge along a gradient of minor differences, it is possible that the sum of these measurements produces discrete functions. Additionally, it is possible that although simultaneous sampling of modalities does not show alignment, temporal dynamics of epigenetics preceding and influencing gene expression may explain the subsequent development of other cell properties. In this model, individual modules are required in specific times and places to create the next developmental process but dwindle once that process is set in motion. This model may explain why gene expression is both dynamic and more striking in its differences across areas during developmental timepoints. Together, this sequential emergence of diverse cell properties and the gradients of these properties across cortical areas may ultimately explain how the blurry lines from each measurement ultimately describe the emergence of cells with distinct functions.

## *In vitro* Human Model Systems as a Framework for Investigating Area-Specific Neurodevelopmental Disorders

Ultimately, multi-omic cortical landscapes can elevate investigations of the signaling pathways that build cortical areas. In addition to addressing century-old questions in neurodevelopment, increasingly comprehensive profiles of the cortex can dissect how this modular network is dismantled in neurodevelopmental and psychiatric disorders. Numerous studies have probed these diseases using adult rodent models (Del Pino et al., 2018; Möhrle et al., 2020). However, transcriptomic comparisons between the human and mouse cortices suggest that a reliance on animal models provides an incomplete view of cortical arealization. These lines of evidence have indicated a lack of conservation between key components of human versus murine cortical patterning, including in the developmental timing of embryonic gene networks (Xue et al., 2013), the genes that drive cell type specification (Hodge et al., 2019), and transcriptomic heterogeneity within cell types (Hodge et al., 2019; Bakken et al., 2021).

Profiles of the developing and adult human cortex serve as a more accurate reference to be compared with post-mortem patient brains, revealing how dysregulated networks within and across cortical areas culminate in neurological pathologies. Recent studies have already begun to survey cortical areas in neurodevelopmental and psychiatric disease states. For example, bulk transcriptomics have been used to interrogate the splicing events and transcriptomic changes in neurotypical brains versus those with neurological disorders (Wu et al., 2017). More recently, the integration of spatial transcriptomic profiles of the human dorsolateral PFC with transcriptomic datasets from neuropsychiatric patient brain samples have revealed layer-enriched expression of genes associated with autism and schizophrenia risk (Maynard et al., 2021). Continued improvements in cortical profiling of both physiological and disease states demonstrate exciting potential in pinpointing the gene networks and cell types directly responsible for area-specific phenotypes of neurological disorders.

More tractable model systems have also emerged to study early stages of the human brain and how it is disrupted in neuropsychiatric disease. With the advent of human embryonic and induced pluripotent stem cell-derived 2D cell cultures and 3D organoids, cell culture systems can more accurately recapitulate gene expression programs of human prenatal neocortex development (Camp et al., 2015; Pollen et al., 2019; Velasco et al., 2019). This is particularly useful in improving our mechanistic understanding of neurodevelopmental disease, as dysregulation between cortical regions and their subcortical connective partners have been implicated in disorders including autism and schizophrenia (Chen et al., 2011; Woodward et al., 2012; Willsey et al., 2013). Unlike primary tissue, two- and three-dimensional cell cultures are particularly well-suited to screen for gene perturbations that result in aberrant cortical arealization or conferred disease risk. Models derived from induced pluripotent stem cells are particularly advantageous in that they can reflect the genetic makeup of patients with neurological disorders. These models involve the reprogramming of adult patient-derived skin or blood cells back into an embryonic, pluripotent state, followed by differentiation into cell types such as cortical neurons and glia. The stem cell culture system also allows us to hypothesize how the cortex may develop in isolation, without the input of extrinsic molecular cues from neighboring regions to guide areal patterning. For example, assembloids, models that fuse organoids that have been differentiated into various tissue types, can help address what extrinsic cues are required for specification of cortical areas such as V1 and PFC. Sergiu Pasca’s lab originally pioneered this method to study neuronal migration of ganglionic eminence-derived GABAergic interneurons that populate the cortex during development (Birey et al., 2017). However, this bottom-up system is widely applicable to the many open questions surrounding cortical arealization. The fusion of differentiated organoids resembling cortical and subcortical regions, for instance, can reveal the reciprocal influence of each area on its neighbor. Coupled with multi-omics, these cell culture systems can reverse engineer intrinsic and extrinsic areal patterning mechanisms and elucidate how these birth the cortical modular network.

## Conclusion

Neurodevelopmental views of cortical arealization have been revolutionized by emerging technologies including bulk transcriptomics, scRNA-seq, and other omics methods. Transcriptomic profiling has revealed that subtle gene expression gradients with dynamic changes in their expression patterns eventually produce discrete functional cortical areas. However, these studies have also underscored that transcriptomics alone is insufficient to understand the spatial and temporal cues that ultimately configure the adult cortex. Instead, genomic profiles need to be integrated with epigenetic and phenotypic assays. Such multi-omic analyses can uncover the relationship among the molecular, structural, and functional characteristics of cell types and areas underlying the development of the cortical modular network. These efforts will increase the depth and resolution of the human cortical landscape, advancing both our understanding of normal development as well as therapeutic strategies for neurodevelopmental and psychiatric diseases.

## Author Contributions

AB and PN conceived of the manuscript. PN, CN, JM, and AB wrote and edited the review. All authors contributed to the article and approved the submitted version.

## Conflict of Interest

The authors declare that the research was conducted in the absence of any commercial or financial relationships that could be construed as a potential conflict of interest.

## Publisher’s Note

All claims expressed in this article are solely those of the authors and do not necessarily represent those of their affiliated organizations, or those of the publisher, the editors and the reviewers. Any product that may be evaluated in this article, or claim that may be made by its manufacturer, is not guaranteed or endorsed by the publisher.
